# Advances in Idiosyncratic Drug-Induced Liver Injury Issues: New Clinical and Mechanistic Analysis Due to Roussel Uclaf Causality Assessment Method Use

**DOI:** 10.3390/ijms241310855

**Published:** 2023-06-29

**Authors:** Rolf Teschke, Gaby Danan

**Affiliations:** 1 Department of Internal Medicine II, Division of Gastroenterology and Hepatology, Klinikum Hanau, Academic Teaching Hospital of the Medical Faculty, Goethe University Frankfurt/Main, Leimenstrasse 20, D-63450 Hanau, Germany; 2 Pharmacovigilance Consultancy, Rue des Ormeaux, 75020 Paris, France; gaby.danan@gmail.com

**Keywords:** DILI, RUCAM, immune systems, genetics, COVID-19, clinical DILI, mechanistic DILI, molecular DILI, top drugs DILI, drug-induced liver injury, herb-induced liver injury, HILI, Roussel Uclaf Causality Assessment Method, original RUCAM, updated RUCAM

## Abstract

Clinical and mechanistic considerations in idiosyncratic drug-induced liver injury (iDILI) remain challenging topics when they are derived from mere case narratives or iDILI cases without valid diagnosis. To overcome these issues, attempts should be made on pathogenetic aspects based on published clinical iDILI cases firmly diagnosed by the original RUCAM (Roussel Uclaf Causality Assessment Method) or the RUCAM version updated in 2016. Analysis of RUCAM-based iDILI cases allowed for evaluating immune and genetic data obtained from the serum and the liver of affected patients. For instance, strong evidence for immune reactions in the liver of patients with RUCAM-based iDILI was provided by the detection of serum anti-CYP 2E1 due to drugs like volatile anesthetics sevoflurane and desflurane, partially associated with the formation of trifluoroacetyl (TFA) halide as toxic intermediates that form protein adducts and may generate reactive oxygen species (ROS). This is accompanied by production of anti-TFA antibodies detected in the serum of these patients. Other RUCAM-based studies on serum ANA (anti-nuclear antibodies) and SMA (anti-smooth muscle antibodies) associated with AIDILI (autoimmune DILI) syn DIAIH (drug-induced autoimmune hepatitis) provide additional evidence of immunological reactions with monocytes as one of several promoting immune cells. In addition, in the blood plasma of patients, mediators like the cytokines IL-22, IL-22 binding protein (IL-22BP), IL-6, IL-10, IL 12p70, IL-17A, IL-23, IP-10, or chemokines such as CD206 and sCD163 were found in DILI due to anti-tuberculosis drugs as ascertained by the prospective updated RUCAM, which scored a high causality. RUCAM-based analysis also provided compelling evidence of genetic factors such as HLA (human leucocyte antigen) alleles contributing to initiate iDILI by a few drugs. In conclusion, analysis of published RUCAM-based iDILI cases provided firm evidence of immune and genetic processes involved in iDILI caused by specific drugs.

## 1. Introduction

Idiosyncratic drug-induced liver injury (iDILI) is a scientific, clinical, pharmacological, toxicological, and regulatory challenge that deserves analytical efforts to unravel its characteristic features based on robust data derived from patients with complete data sets and following a mandatory causality assessment [1,2,3]. Within the last few years, substantial advances in the field of iDILI were recognized [1], supported by 81,856 worldwide clinical iDILI cases [4], all of which have been assessed for causality by the original RUCAM (Roussel Uclaf Causality Assessment Method) published in 1993 [5], or the now preferred updated RUCAM published in 2016 [6]. A careful causality assessment is stringent since many suspected iDILIs were not caused by drugs but by alternative causes [7,8].

Several scientists from the US [3] and Europe [9,10] highly appreciated the scientometric evaluation of DILI knowledge worldwide with data comprising 1995 publications from 79 countries and regions [11]. This report was refreshing because the authors conducting the study were not affiliated with any Western network but came from China. The authors carefully analyzed the worldwide knowledge base on idiosyncratic DILI, listed several rankings, presented details viewed as promotion of RUCAM use, and described a positive trend of DILI reports for each year between 2010 and 2019. They also assumed that in 2020, nearly 340 published DILI studies will be available [11] in line with an increase of reports on DILI and HILI cases with assessments by the RUCAM for causality [4]. The scientometric study confirmed the high worldwide interest in DILI publications but missed details on the causality assessment method used for individual drugs causing the DILI [11]. This study also showed the top 10 countries involved in DILI research; they include the US, China, Japan, Germany, UK, Spain, France, the Netherlands, Sweden, and Canada. Most informatively, various aspects of DILI were comprehensively analyzed and discussed, considering preferential definition of criteria, global incidence rates, clinical features, or pathogenetic considerations including the role of immunology, the control of cell death pathways, susceptible HLA (Human Leukocyte Antigen) identification, or best causality assessment criteria and methods, all topics that were considered as the knowledge base for DILI research [11]. On a promising note, the RUCAM of 1993 [5] was highlighted as a report that was often co-cited (n = 256) and ranked first in the category of the top 10 co-cited references related to DILI research [11]. The Chinese study also listed two authors from the University of Michigan and Frankfurt/Main, who may have significant influence on DILI research with more publications (n = 46; n = 39) and co-citations (n = 382; n = 945), which was viewed as encouraging data according to the Chinese authors. Investigators from the US were described as the largest group since most of their publications were derived from a US network, whereas another investigator from the University of Frankfurt/Main was correctly described as not being part of any network [11], confirming scientific and financial independence from any pressure within circles related to DILI. Clearly, the promotional independent Chinese scientometric evaluation by external scientists is recommended reading for other DILI experts, because it calls for performing more DILI studies. 

RUCAM is preferable to other causality assessment methods (CAMs) because it has been validated with cases including positive rechallenge. The other methods were not validated at all. Since 1993, RUCAM has been broadly used over the world by experts in DILI. The quality of RUCAM based DILI cases depends on the quality and completeness of the provided cases and the qualification of the submitting physician. 

The present article summarizes and evaluates recent advances of iDILI with a focus on new clinical and mechanistic aspects based on cases with verified diagnosis using the RUCAM.

## 2. Search Terms and Strategy

The literature search strategy involved the PubMed database and Google Science, focusing on these keywords: idiosyncratic drug-induced liver injury; RUCAM; mechanistic steps; immune systems; genetics; and combinations thereof. Around 89,900,000 articles were provided for the term of drug-induced liver injury challenges, 148,000,000 publications for the term drug-induced liver injury, and 238,000,000 hits for the term of DILI. Then, the initial fifty reports of the three groups were analyzed for their possible inclusion in this article. The search was started on 30 October 2022, and completed on 31 January 2023. Publications were complemented by the large private archives of the authors. There was a restriction on publications in English.

## 3. New RUCAM-Based iDILI Cases

Numerous review articles, case series, and single case reports around the world described new drugs implicated in iDILI, but clinical features often remained vague and controversial when a robust causality assessment method such as the RUCAM was not applied [1,2,3]. These shortcomings also apply to the LiverTox database by presenting cases of assumed DILI not evaluated by any causality assessment including RUCAM [12].

Publication details of new iDILI cases by known drugs were informative. Such cases now present RUCAM-based causality associated with individual causality gradings and a selection of implicated drugs listed in alphabetical order [3]: amlodipine (RUCAM score 6, probable causality grading) [13], anastrozole (score 6, probable) [14], atorvastatin (score 9, highly probable) [15], atovaquone (score 9, highly probable) [16], candesartan (score 8, probable) [17], ciprofloxacin (score 11, highly probable) [18], fenofibrate (score 10, highly probable) [19], flucloxacillin (score 8, probable) [20], gemcitabine (score 10, highly probable) [21], infliximab (score 10, highly probable) [22], metamizole (median score 7, probable) [23], and teriflunomide (score 8, probable) [24]. Probable and highly probable RUCAM-based causality gradings commonly reflect complete case data sets by early prospective collection of the required clinical and laboratory data [3] in line with previous recommendations [6]. Such high-graded iDILI cases are also suitable for addition to the 81,856 cases published until mid-2020 [4] and for inclusion in the LiverTox database, replacing other poor-quality cases not assessed by the RUCAM [12].

Advances and breakthroughs in the RUCAM field were also noted because for drugs which are implicated in iDILI, the updated RUCAM was increasingly used and mentioned for reasons of transparency in the title of virtually all publications [25,26,27,28,29,30,31,32,33,34,35,36,37,38,39,40,41,42,43]. Examples are case series with multiple drugs [25,26,27,28,29,30,31,32,33] as well as individual drugs in alphabetical order: androgenic anabolic steroid drugs (updated RUCAM score 6, probable causality grading) [34], atezolizumab (score 7, probable) [35], fluoroquinolones (scores 6–8, probable, and scores ≥9, highly probable) [36], methotrexate (scores 6–8/≥9, probable and highly probable) [37], nevirapine (scores 6–8, probable) [38], para-aminobenzoate (score 10, highly probable) [39], rosuvastatin (score 9, highly probable) [40], pazopanib (score 8, probable) [41], teriflunomide (score and causality grading not reported) [42], and tigecycline (scores as mean ± SD: 6.8 ± 0.7, probable, and 9.1 ± 0.3, highly probable) [43]. Analysis of the nine reports above showed that iDILI cases with a possible causality grading have also been included in a few of these publications, which should not be carried out because it clouds the robust clinical features provided by iDILI cases, for which probable and highly probable causalities were found. The updated RUCAM is now the preferred approach for evaluation of iDILI cases because it considers elements such as exclusion of HEV (hepatitis E virus) as mandatory, quantifies the gender-based consumption of alcohol, and defines liver injury by thresholds of ALT (alanine aminotransferase) activities ≥5 times the upper limit of normal (ULN) or ALP (alkaline phosphatase) activities ≥2 times the ULN [6], items not included in the original RUCAM [5]. Of note, many earlier publications from DILI registries, networks, or databases used lower ALT and ALP thresholds, which erroneously included cases of liver adaptation [10,44,45], and left exclusion of HEV infections unconsidered [44,45] or optional [46]. These omissions call for caution with interpretation of published results [10], now avoidable by using the updated RUCAM [6]. Problems remain with the LiverTox database, classified as a paradox because of gaps between promising DILI case data with causality assessment using the RUCAM and the reality of missing RUCAM data, which became a matter of debate making the information from this database questionable for clinical or scientific use [12].

In general, there are several excellent publications on DILI, which lack case evaluation using the RUCAM and provide results with a cautionary conclusion. As an example, in a recent article on iDILI caused by protein kinase inhibitors for cancer, the case narratives forgot to analyze and discuss alternative causes and polymedication common in this special cohort [47], but it was published with the use of the RUCAM [35,41,48]. Alternative cases are frequently found in DILI cohorts assessed with the RUCAM [7,8] but often ignored leading to inappropriate description of case features [12].

The increased publication rate of RUCAM-based DILI cases [3] is encouraging, in line with the trend observed since 1993 [4], but now with more focus on the updated version of the RUCAM at the expense of the original RUCAM [25,26,27,28,29,30,31,32,33,34,35,36,37,38,39,40,41,42,43]. This improves the chances that future cases may provide additional mechanistic immune and genetic data and help clarify molecular idiosyncratic toxicology of drugs in humans [48].

## 4. RUCAM in DILI of COVID-19 Patients

Abnormal liver tests (LTs) were frequent findings among patients experiencing infections by COVID-19 (coronavirus disease-2019), attributed to RUCAM-based iDILI in this polymedicated cohort (Table 1) [49,50,51,52,53,54,55,56].

Apart from the eight reports (Table 1), several cases of COVID-19 patients with drug treatment and documented increased LTs were published but were not assessed for DILI by using the RUCAM and were not evaluated for non-drug causes [57,58]. Among the eight publications presenting RUCAM-based DILI cases (Table 1) [49,50,51,52,53,54,55,56], one of these [49] used the original RUCAM of 1993 [5] whereas the applied RUCAM version was not disclosed in two other reports [52,53]. In contrast, the updated RUCAM was used in the remaining five reports [50,51,54,55,56], which was a better approach as the updated RUCAM should now be preferred [6]. DILI cases with a low RUCAM-based causality grading are not often submitted for consideration of publication, because they are easily declined already at time of submission or later after careful evaluation by reviewers. In virtually all previous reports, case data were collected retrospectively [57,58], providing incomplete information to some extent, not fulfilling requirements of high RUCAM-based causality gradings needed for publication in reputed journals. To overcome these problems, prospective studies are urgently needed to facilitate proactive collection of complete data sets [57].

Summarizing the most important results obtained from the eight publications (Table 1), which cover overall 465 COVID-19 patients with RUCAM-based iDILI cases published 2020–2022 [49,50,51,52,53,54,55,56], a detailed description of clinical features is feasible [58]: (1) the male gender prevailed compared with females; (2) age was in a range from 45 to 57 years; (3) hepatocellular injury was more commonly observed than cholestatic or mixed injury; and (4) polymedication is likely a risk for liver injury in the COVID-19 cohort characterized by concomitant use of many drugs for treating multimorbidity. The existence of iDILI in a COVID-19 cohort will inevitably confound the clinical features of COVID-19 if not differentiated from each other.

Mechanistic steps of drugs implicated in RUCAM-based DILI have been proposed in several of the eight reports [49,50,51,52,53,54,55,56]. For instance, DILI by tocilizumab (TCZ), a humanized recombinant monoclonal antibody with properties as an IL-6 receptor antagonist against the cytokine storm, may be initiated by its firm binding to IL-6 receptors [49]. Other considerations include multipharmacy, earlier therapy with drugs known for their potential of causing liver injury, drug–drug interactions, and inhibition or induction of drug-metabolizing enzymes, whereby drugs like lopinavir/ritonavir could have triggered the development of liver injury by TCZ [57]. Based on a large case series, nonalcoholic fatty liver disease (NAFLD) was suspected as a risk factor for the liver injury by drugs [50]. Because NAFLD is commonly associated with overweight and obesity, both of which exert induction of hepatic microsomal cytochrome P450 (CYP) 2E1, a possible causal role of CYP 2E1 in iDILI of COVID-19 patients can be assumed. In addition, there was also a focus on molecular interactions connected to CYP 3A4, strongly inhibited by ritonavir, which possibly promotes the liver injury caused by azithromycin through mechanisms at the level of the CYP molecule [51]. Molecular interactions causing DILI during inflammation could also be accompanied by production of ROS (reactive oxygen species) within inflammatory cells, possibly through myeloperoxidase, an enzyme found in inflammatory cells like macrophages and neutrophils, while additional immune mechanisms were assumed in a small subset of DILI cases [51]. For the liver injury caused by dabigatran, an idiosyncratic type of liver injury was assumed rather than an intrinsic one [52]. The DILI by favipiravir or its metabolites also was ascribed to an idiosyncratic reaction [53]. Not to be neglected, continuous drug use can cause self-inhibition of liver metabolism, which may enhance the favipiravir/inactive metabolite ratio, assumed as a risk factor for the injury, like a high drug intake [53]. According to another proposal, a high loading dose of a drug associated with the use of potentially hepatotoxic drugs may facilitate the liver injury [54]. Limited to only two analgesic-antipyretic drugs, ibuprofen and acetaminophen, no mechanistic proposals were made [55]. Mechanistic steps were not presented by another study with many drugs [56]. Proposals which were not based on iDILI cases assessed for causality using the RUCAM must be observed with caution [59,60,61,62,63]. As an example, a report claimed an increased risk of iDILI by a factor of four if lopinavir is used together with ritonavir [59].

Mechanistically, the liver injury by hydroxychloroquine use was causally related with the generation of reactive metabolites and oxidative stress induced by this drug or based on some idiosyncratic and/or synergistic effect associated with inflammatory processes caused by the infection [60]. Among various liver injury mechanisms, oxidative stress was proposed for iDILI by azithromycin, hydroxychloroquine, or lopinavir/ritonavir [61]. To verify mechanistic proposals for the drugs of interest implicated in the liver injury (Table 1), additional evidence to be derived from respective COVID-19 patients with iDILI assessed by the updated RUCAM must be provided to reduce speculation.

The RUCAM was used smoothly in almost 100,000 cases of iDILI and HILI (herb-induced liver injury) [4] and many other cases, as well as in iDILI found in COVID-19 patients (Table 1) [50,51,52,53,54,55,56], a success likely attributed to a stepwise approach provided in earlier publications [5,6] and subsequently through clear procedural instructions on how best to use the updated RUCAM [62]. Suggestions of possible improvements in practice during the regular use of the updated RUCAM were provided in a report on the determination of causality in DILI patients with COVID-19 clinical syndrome, described in a cohort of 72 COVID-19 patients with suspected DILI [56]. Two independent rating pairs (consisting of two clinical pharmacologists plus two general physicians), who had received a short training program for pilot testing just prior to the actual RUCAM use, determined the likelihood of DILI using the RUCAM scale in 72 DILI patients. As a result, the Krippendorf *kappa* was 0.52, with an intraclass correlation coefficient (ICC) of 0.79, which was viewed by the authors as excellent reliability for using the updated RUCAM [56]. Whether this is achieved through the prior training remains to be verified by a group of assessors without prior training. The good reliability results obtained now by external validation confirm a very high interrater agreement of an earlier report analyzing its own external validation of RUCAM use [45]. This result was remarkable as the data of a cohort with 72 patients were retrospectively collected, which usually provides poor case data quality as described [56] and noted earlier as asking to use a prospective study design to reach high RUCAM-based causality gradings due to data completeness [6,57]. Of note, for any new method, a short training program should be completed before the evaluation, which is self-evident and therefore not explicitly mentioned among the general recommendations on how best to use the updated RUCAM [62]. Promoting was the expert note that the harmonization of DILI causality tools through the introduction of the original RUCAM and its updated version has resolved evident uncertainties [56], in line with previous proposals [63]. Worldwide harmonization of RUCAM use is in good progress, shown by the 81,856 DILI cases published up to mid-2020 [4] alone, outperforming any other tool regarding case numbers [64] including electronic modifications of the RUCAM that have the problem of correct internal method validation and lack any external validation [62,65].

## 5. Top Drugs Involved in RUCAM-Based iDILI

Using some reports as examples [44,45,66,67,68,69,70,71,72,73,74,75,76,77], a valid compilation of worldwide top drugs causing DILI with diagnosis verified by the RUCAM is available (Table 2) [13].

RUCAM of 1993 was used by the groups of Andrade [45,67], Björnsson [44,71,73], Devarbhavi [69], Douros [74], García-Cortés [68], Lucena [70], Rathi [77], Robles-Días [75], Stephens [72], Wai [66], and Zhu [76], who provided RUCAM-based cases with proper diagnosis enabling the list of top drugs causing DILI (Table 2), which was encouraging. The top of the 10 drugs most implicated in causing iDILI was amoxicillin-clavulanate with 333 published RUCAM-based DILI cases, followed by flucloxacillin, atorvastatin, disulfiram, diclofenac, simvastatin, carbamazepine, ibuprofen, erythromycin, and anabolic steroids (Table 2). This ranking was established after analysis of worldwide reported publications comprising case reports, case series, and drugs of DILI registries (Table 1) [44,45,66,67,68,69,70,71,72,73,74,75,76,77] and can replace several top rankings of drugs causing DILI with cases restricted to only a single country.

However, it is discouraging to see a new recent policy of switching from DILI assessment using the RUCAM [45,71,73] to fragile non-RUCAM evaluation of drugs found in the US LiverTox database, which is attempting to rank top drugs most implicated in causing DILI based on the number of published DILI reports of individual drugs [78]. In other words, a high probability association of DILI is constructed by means of a high case number, an attempt to provide support for the authors of the LiverTox database [79]. This approach is questionable [78,79] as critically discussed [10,12] in the face of up to 47% of cases with suspected DILI that must be attributed to non-drug causes [7,8,80,81] confounding the diagnosis of DILI contained in the LiverTox database [78,79]. The RUCAM helps describe valid clinical features of DILI [1,2,3,4,13,14,15,16,17,18,19,20,21,22,23,24,25,26,27,28,29,30,31,32,33,34,35,36,37,38,39,40,41,42,43,49,50,51,52,53,54,55,56,57,58,82] to be used, for instance, for the LiverTox database to ensure robust details on potentially hepatotoxic drugs.

## 6. Advances in Wide Use of the RUCAM Assessing Causality in DILI Cases

**Figure 1:**
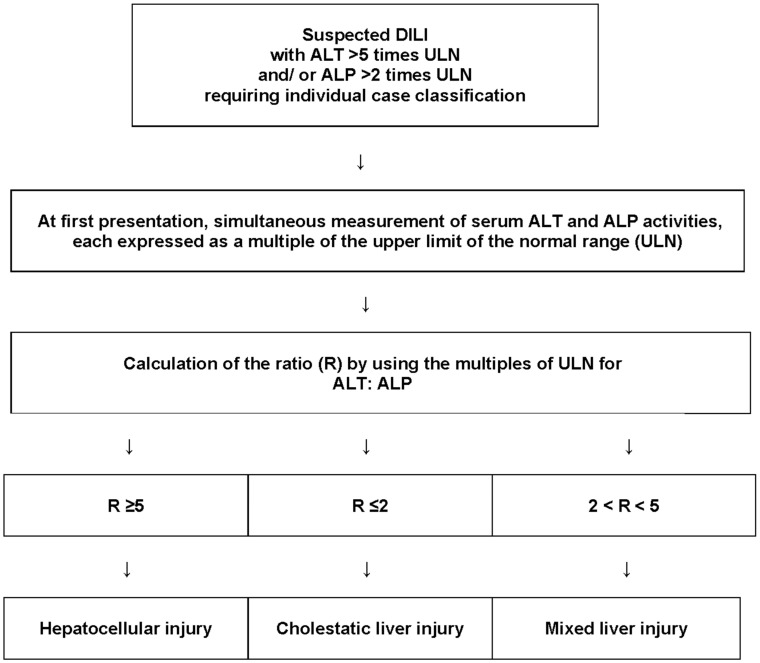
Classification of the liver injury pattern. The determination of the individual liver injury pattern is required to assess causality in suspected DILI cases by the updated RUCAM that exists with two versions; one is destined for the hepatocellular injury, and the other one for the cholestatic liver injury/mixed liver injury. The approach is identical for suspected herb-induced liver injury (HILI). Adapted from a previous open access publication [6]. Abbreviations: ALP, alkaline phosphatase; ALT, alanine aminotransferase; DILI, drug-induced liver injury; ULN, upper limit of normal; R, ratio.

The worldwide use of the RUCAM is well documented by 81,856 DILI cases evaluated by the RUCAM to verify the diagnosis through causality assessment and published from 1993 to mid-2020 with increasing tendency [4]. The RUCAM outperforms by far regarding published DILI case number all other causality assessment methods [64]. The appreciation and popularity of the RUCAM can be traced back to its clear diagnostic algorithm [5,6,9,11,62,64,83], based on principles of Artificial Intelligence (AI) [84], its perfect method of validation using positive exposure tests of published cases as a commonly accepted gold standard [85]. Other promoting features include its specificity for liver injury, defining typical elements of the liver injury associated with a scoring system that ensures objectivity and allows through addition of the individual scores the gain of a final score with specific causality levels: score ≤ 0, excluded causality; 1–2, unlikely; 3–5, possible; 6–8, probable; and ≥9, highly probable [5,6]. With its scoring algorithm, the quantifying RUCAM surpasses by far any other non-quantifying causality assessment method like the global introspection approaches or so-called expert opinion methods, all of which provide objective results and are, by definition, not suitable for method validation, disregarding these as gold standard methods [6,64]. The RUCAM is also appreciated for its transparency [5,6], user-friendly application [4], perfect handling of concomitant use of multiple potential hepatotoxic drugs [28,29,31], reproducibility with good interrater performance [45,56], and defining criteria of different liver injury patterns such as hepatocellular liver injury, cholestatic liver injury, or mixed liver injury, first published already in 1993 [5] and mentioned again in 2016 [6], now shown in a flow chart (Figure 1).

This classical differentiation of liver injury pattern, known also as phenotypes, is used in most DILI reports, although occasionally without quoting the source. It is mandatory for causality assessment using RUCAM but also helpful for defining clinical DILI features. As the determination of the liver injury pattern requires only the results of serum ALT and ALP activities (Figure 1), this approach saves financial resources and does not require an invasive and risky liver biopsy. The classification is essential for using the RUCAM in iDILI cases destined to establish pathogenetic mechanisms.

## 7. Progress of Molecular and Mechanistic Immune Mechanisms in iDILI

Firm data on molecular processes involved in idiosyncratic DILI as well as accurate mechanistic steps leading to the liver injury are fragmentarily found in most related publications. Many proposals were interesting but purely speculative lacking any evidence base, while others were derived from considerations based on circumstantial evidence only [86,87,88,89,90,91,92,93,94,95,96,97,98,99,100,101,102,103], leaving many unresolved basic issues [103]. Several of these publications provide graphical abstracts or schematic presentations on mechanistic pathways with contradictory illustrations, clouding mechanistic issues. Abundant results derived from studies using animal models have been published that were viewed as unsuitable for translation to human diseases like iDILI [104,105]. The basic problem is the previous lack of using RUCAM-based iDILI with verified diagnosis that would allow for more evidence-based data on most molecular and mechanistic aspects in clinical iDILI. Appropriate pathogenetic studies are preferentially restricted now to analytical data obtained in fluids like blood or urine of patients with iDILI verified by using the RUCAM, rarely also from liver histology evaluation, and analyzed for their potential to be used as strong evidence-elucidating pathogenetic features related to immunology systems and genetics [105,106,107,108,109].

### 7.1. Serum Anti-Cytochrome P450 Antibodies

**Figure 2:**
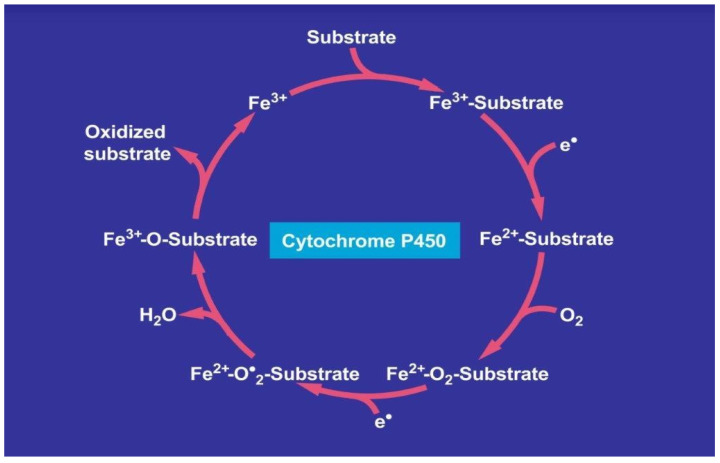
Catalytic CYP cycle involved in hepatic drug metabolism. Cytochrome P450 stands for its various isoforms. The figure was adapted from recent open access reports [105,109].

Cytochrome P450 (CYP) with its various isoforms is found in the microsomal fraction of the liver cells that correspond to the smooth endoplasmic reticulum visible as electron microscopy study [109]. It is involved in the hepatic metabolism of most drugs to harmless chemicals, in rare instances. However, CYP promotes the generation of toxic metabolites responsible for iDILI [105,109] by a sequence of events carried out as a catalytic cycle (Figure 2).

In comparison to many other substrates, drugs enter the catalytic CYP cycle as substrate, as shown on the top of the cycle, whereby drugs bind to CYP (Figure 2). The subsequent events follow a multi-step process. Finally, the drug leaves the CYP cycle after it is oxidized forming now as a metabolite. Mechanistically, the first electron is provided to CYP by NADPH + H^+^ via the NADPH CYP reductase, whereby the reduced form of CYP with Fe^2+^ is generated, which finally becomes oxidized again after splitting off the oxidized substrate. Then, CYP becomes free again for the next substrate to be oxidized (Figure 2) [105,109]. Through introduction of molecular oxygen, a multi-compound reactive complex is generated, a process facilitated by inclusion of another electron that commonly is provided through the NADPH CYP reductase or a similar but NADPH independent reductase.

Among the drugs implicated in triggering iDILI, 58.3% are metabolized by CYP isoforms, whereas the remaining drugs undergo metabolism through other pathways [103]. It is fascinating that the clinical observation that the use of some of the drugs, which are metabolized by CYP isoforms, leads to the production of antibodies against cytochrome P450 (CYP), found in the serum of patients with iDILI. This is a perfect example of how information of intrahepatic immune processes connected with iDILI are released in the blood, ready to be analyzed for pathogenetic considerations related to immunology issues [106,107,108,109]. In this context, serum anti-CYP 2E1 antibodies were detected following use of the volatile anesthetic sevoflurane in four patients with iDILI and verified diagnosis by using the RUCAM, which led to highly probable causality [106]. These results were confirmed by a subsequent study of sevoflurane and desflurane, another modern volatile anesthetic, whereby sevoflurane was applied mostly alone and rarely combined with desflurane [107]. Five patients with iDILI reached a RUCAM score of ≥6, and serum anti-CYP 2E1 antibodies were found in three patients with scores of 12, 7, and 6, while in two patients with a score of 12 and 7, respectively, no antibodies were detected. Additional data of the anesthetic cohorts are available (Table 3).

Only part of the RUCAM-based iDILI cases were associated with these antibodies, while additional analysis revealed that anti-CYP 2E1 antibodies were detected unexpectedly in patients who were exposed to the anesthetics but did not fulfill the RUCAM criteria of iDILI [107], thereby not providing a homogenous antibody picture. In support of external RUCAM validation, this study showed again an excellent interrater performance [107] confirming previous reports [45,56].

Like sevoflurane and desflurane, cases of liver injury associated with serum anti-CYP antibodies due to use of other drugs are under discussion [108]. Among these were halothane (causing anti-CYP 2E1 antibodies) [102,109,110,111,112,113,114], isoflurane (anti-CYP 2E1) [114], isoniazid (anti-CYP 2C9) [109], as well as dihydralazine (anti-CYP 1A2), tienilic acid (anti-CYP 2C9), and antiepileptics (anti-CYP 3A) [102].

The association of serum anti-CYP isoforms with iDILI by some drugs suggests an immunological involvement in this process but not necessarily a causal immune association leading to the liver injury. In fact, liver injury by drugs such as sevoflurane and desflurane is also associated with the formation of trifluoroacetyl (TFA) halide as toxic intermediates [106,107] that form protein adducts and may generate free radicals, known as reactive oxygen species (ROS) [106,115,116]. This is accompanied by anti-TFA antibodies detected in the serum of some but not all patients with liver injury by volatile anesthetics (Table 3) [106,107].

Serum anti-CYP antibodies in connection with iDILI were reported only for a few CYP-dependent drugs, leaving aside many of the drugs metabolized by CYPs that do not generate these antibodies for unknown reasons. The lack of antibody data can be real; alternatively, no comprehensive analytical approaches were performed. To solve this issue, future studies should focus on detection of serum anti-CYP antibodies, considering specifically drugs metabolized by CYPs and causing iDILI with valid diagnosis ascertained by the updated RUCAM, with focus on high RUCAM-based causalities of probable or highly probable.

### 7.2. Serum Anti-Nuclear Antibodies and Anti-Smooth Muscle Antibodies

Anti-nuclear antibodies (ANA), anti-smooth muscle antibodies (ASMA), and rarely other autoantibodies that might be detected in the serum of patients treated with conventional drugs who experienced liver injury assessed for causality by RUCAM [117,118]. For instance, among a cohort of 139 RUCAM-based iDILI patients, serum ANA results were positive in 95 patients (68.3%) and negative in 44 patients (32.7%), but data remain open for discussion since cases with a possible causality grading were included, herbal and dietary supplements were among the DILI patients, and the original RUCAM was used lacking exclusion of HEV rather than the updated RUCAM [117]. In 71% of these cases, ANA and/or SMA titers were positive. In the other earlier RUCAM-based study, similar data were reported in addition to normal values of immunoglobulins IgA, IgG, and IgM [118]. These two studies on serum ANA and SMA provide evidence of immunology reactions in the liver of some but not all patients with iDILI.

### 7.3. Specifics of Hepatic Immunology

Direct rather than circumstantial evidence for a participation of the innate and adaptive immune systems in iDILI with RUCAM-based verification of the diagnosis is increasingly observed, although there were still narratives published on this topic that come along without the RUCAM. The initiation of an immune response in emerging iDILI likely requires the activation of antigen presenting cells (APCs) by different molecules including danger-associated molecular pattern molecules (DAMPs) [89]. Direct evidence for the role of the innate immune system in causing the iDILI was convincingly shown for offending drugs such as diclofenac, indomethacin, levofloxacin, and phencoumon through studies of monocyte-derived hepatocyte-like cells in iDILI cases assessed by the updated RUCAM [119]. These findings support the concept that monocytes are part of the innate immune system [48,89,120,121,122]. Going back to the origin, hepatic monocytes are commonly derived from bone marrow progenitors, and when released into the blood, they can enter the liver, where they differentiate into liver resident macrophages like Kupffer cells (KCs) and infiltrating monocyte-derived macrophages (MoMF), allowing for crosstalk with hepatic monocytes within the liver and intensive exchange of inflammatory mediators [122]. Using commercially available kits, they are detectable in the blood of iDILI patients as circulatory mediators such as the cytokines IL-22, IL-22 binding protein (IL-22BP), IL-6, IL-10, IL 12p70, IL-17A, IL-23, IP-10, or chemokines like CD206 and sCD163; examples were patients with the diagnosis of suspected iDILI by anti-tuberculosis drugs and verified by the prospective use of the updated RUCAM that provided high causality gradings [123]. The parameters IP-10 and sCD163 are usable as risk factors of future cases of this DILI entity. More robust data on circulatory mediators in the serum of iDILI patients are expected, provided the use of the updated RUCAM verifies the diagnosis.

### 7.4. Drug-Induced Autoimmune Liver Injury versus Genuine Autoimmune Hepatitis

Direct evidence for the role of the hepatic immune system is provided by a DILI subgroup through studies on cases of DIAIH, all assessed for causality using the RUCAM to establish the diagnosis autoimmune DILI caused by several drugs as follows [124]: antimicrobials [125,126], atorvastatin [124], augmentin [125], ceftriaxone [125], diclofenac [127], direct oral anticoagulants [128], hydralazine [127], infliximab [129,130], isoniazid [127], ketoprofen [125], minocycline [127], methyldopa [127], nimesulide [125], nitrofurantoin [127,129,131,132], non-steroidal anti-inflammatory drugs [125,128,131,133], sorafenib [124], and statins [126,128,132]. The studies discussed above provided a clear differentiation of DIAIH from the classical genuine AIH by using scores of the simplified AIH scale for assessing the AIH [134] and applying the RUCAM scores [5,6] for evaluating DIAIH [124]. Similar proposals were made for cases in pediatrics [135], now enforcing the use of the updated RUCAM for suspected DIAIH [6]. Apart from triggering DIAIH, some of these drugs also can cause common DILI-lacking autoimmunity features, as noted by one study [129] and confirming previous statements [89]. Summarized are details of selected DIAIH cases (Table 4).

In line with the immune participation is the fact that DIAIH responds well to the immune modulatory action of glucocorticoids without relapse after treatment cessation, whereas relapse in genuine AIH is a common feature and typical for this specific disease entity [124,128]. However, glucocorticoids are only partially effective in treating patients with unselected idiosyncratic DILI caused by various drugs as a whole DILI cohort, suggesting that only part of the DILI cases were initiated by immune mechanisms [48] according to previous proposals [89]. Direct evidence for an involvement of the immune system in idiosyncratic DILI was also provided by its rare association with the immune-triggered Stevens–Johnson syndrome (SJS) and toxic epidermal necrolysis (TEN) caused by a small group of drugs [121]. In these cases, causality of idiosyncratic DILI was evaluated by the RUCAM and of SJS/TEN by the Algorithm for Drug Causality for Epidermal Necrolysis, which reached highly probable or probable causalities in all cases.

## 8. Advances in Genetics

Already in 2009 and after analysis of iDILI cases with probable and highly probable RUCAM causalities in 92% of cases, direct evidence was presented that iDILI is triggered partly by genetic susceptibility of human leucocyte antigen (HLA) alleles [136]. In this report alongside the Genome-wide association study (GWAS), HLA-B*5701 genotype was determined as a major determinant of iDILI caused by flucloxacillin. Similar results of HLA genotypes were found in other RUCAM-based iDILI cases for several drugs including anti-tuberculosis drugs [137], nitrofurantoin [138,139], amoxicillin-clavulanate [70,72], diclofenac, azathioprine, isoniazid, fenofibrate [139], and flucloxacillin [20,140]. However, there was a lack of data reproducibility with respect to amoxicillin based on iDILI cases evaluated by the RUCAM [141] as well as regarding nitrofurantoin considering iDILI cases evaluated for causality using global introspection, a non-RUCAM approach lacking proper validation and individual element scoring [142].

Interestingly, an assumed HLA association of liver injury by amoxicillin-clavulanate was reported already in 1999, but such data remained vague because cases were assessed for the liver injury pattern only but not for causality using the original RUCAM of 1993 [143]. An important feature was the early recognition to apply the RUCAM for valid causality evaluation in HLA studies, to be viewed as a general recommendation for future studies on this subject [136]. Some subsequent publications also used the RUCAM [20,70,72,137,138,139,140,141], but various other studies abstained from using it and provided preliminary and vague HLA data not based on evidence and not suitable for further consideration. Future prospects in HLA genetics were outlined [144] but must include iDILI cases evaluated by using the updated RUCAM with high causalities. Preferred studies are those with a prospective design that ensures proactive collection of complete case data required for high causality gradings [6]. Retrospective studies can also be evaluated [6,62,83], but incomplete data commonly provide many cases with a merely possible causality as shown in some of the HLA studies [20,70,72,137,138,139,140,141]. These cases should not be included in publications to avoid clouding robust data obtained from cases with a probable or highly probable causality grading.

More data are needed to close the gaps between HLA data and pathogenetic aspects of iDILI [144]. Current HLA data are a little step forward to partially characterize iDILI [20,70,72,136,137,138,139,140,141,144], but as genetic markers, they struggled by a high negative predictive value and low positive predictive value, limiting which reduces their values in a clinical setting to prospectively predict iDILI risk [145]. The overall clinical value of HLA B*5701 pre-assessment in an individual patient, for whom a treatment with flucloxacillin is planned, is in question since there is less than 1/500 chance that the patient will develop iDILI in case of HLA B*5701 positivity [89]. Although HLA studies showed an association of genetics with iDILI caused by a limited number of drugs, their contribution in elucidating additional mechanistic details in iDILI remains marginal [144], let alone its value as diagnostic biomarker, preventive risk factor, or causality, as well [89,144,145].

## 9. Metabolomics

Studies on metabolomics in iDILI cases with analysis of metabolites in biological samples like blood or urine seem to become promising tools that could help shed more light on the biological mechanism pathways of liver injury [32,95,146,147,148,149]. However, advances of metabolomics analyses can be expected only if results were derived from RUCAM-based iDILI cases [32,148,149] that followed the global use of the original and updated RUCAM [4,5] in line with the balanced view and appreciation of the RUCAM [1]. Consensus exists that further investigations in patients with iDILI of high RUCAM causalities are essential in searching for mechanistic steps [103,149].

Metabolomics studies could be helpful in iDILI cases assessed by the updated RUCAM with focus on drugs like isoniazid, diclofenac, azathioprine and other thiopurines, ciprofloxacin and other fluroquinolones, atorvastatin and other statins, nimesulide, interferon beta, and fasiglifam that lack detectable HLA association [144]. A focus could also be on drugs like amoxicillin-clavulanate, allopurinol, azathioprine/6-mercaptopurine, floxuridine, hydralazine, infliximab, interferon alpha/peginterferon, interferon beta, methotrexate, minocycline, nitrofurantoin, pyrazinamide, rifampicin, sodium aurothiomalate, sulfasalazine, and thioguanine that are not metabolized by CYP isoforms or on the many drugs that are not associated with anti-CYP antibodies although they are substrates for CYP isoforms [103].

## 10. Gut Microbiome

Advances in iDILI evaluated for its modulation by the gut microbiome were also promising [32,148,149]. Expanding metabolomic studies to urine analysis combined with iDILI caused by anti-tuberculosis drugs and assessed with the updated RUCAM with high causality gradings revealed 28 major metabolites involved in functions of bile secretion, nicotinate and nicotinamide metabolism, tryptophan metabolism, and ABC (ATP binding cassette) transporters, characterizing metabolic and gut microbiome features and correlating these with clinical data [32]. The emerging role of gut microbiota dysbiosis in iDILI was supported by antibiotics, which enhanced the liver injury as assessed for causality using the RUCAM [149].

## 11. Conclusions

Compelling evidence now exists from studies on RUCAM-based iDILI cases that immunology and genetic features of predisposed patients are partially involved in iDILI caused by a few selected drugs. However, even for this small group of drugs, uncertainty remains on individual steps leading to the liver injury. Moving from mere case narratives with unclear or only circumstantial evidence, future pathogenetic investigations should be based on a prospective study protocol to proactively collect iDILI cases assessed for causality using the updated RUCAM of 2016 retaining data derived from cases with high causality gradings only achieved by prior removing cases with possible causality. This well-designed study cohort should allow for further analytical studies, best done in blood or urine of affected iDILI patients with parameters like blood circulatory inflammatory and genetic mediators of hepatic origin or metabolomics that would reflect possible mechanistic processes in the liver. These quantitative blood parameters could then be correlated with numbers of cells in the liver generating the mediators, to be counted in the liver of patients with iDILI diagnosis based on the updated RUCAM.

## Figures and Tables

**Table 1:** Compilation of COVID-19 patients with RUCAM-based iDILI.

First Author Country Cases (n) Drugs (n)	COVID-19 Patients with RUCAM-Based iDILI
Muhović, 2020 [49] Montenegro (cases, n = 1) (drugs, n = 4)	The case of a male patient with DILI by tocilizumab (TCZ) and COVID-19 infection that caused a cytokine storm is reported [49]. With the original RUCAM [5] instead of the commonly preferred updated RUCAM [6], causality for TCZ was probable based on a RUCAM score of 8. Such high causalities were commonly achieved with complete case data sets that were prospectively asked for at the time DILI was first suspected. TCZ is a humanized recombinant monoclonal antibody, which acts as an IL-6 receptor antagonist through specific binding to IL-6 receptors. Preexisting liver disease was excluded as well as anoxia that might have caused liver hypoxia as a confounding variable. It was noted that slightly elevated transaminases were detected before TCZ hepatotoxicity was observed, conditions similar to other COVID-19 patients with a severe clinical course. Patient’s comedication included azithromycin, ceftriaxone, chloroquine, lopinavir, methylprednisolone, and ritonavir, but none of these drugs were considered as offending drugs implicated in the liver injury, although a contributory role of the previously used antiviral drugs lopinavir/ritonavir is possible.
Chen, 2021 [50] China (cases, n = 830) (discussed drugs, n = 4)	A total of 830 COVID-19 patients with liver injury were analyzed. This is the largest study cohort evaluated for causality [50], using the updated RUCAM [6]. Among 74/830 cases, the RUCAM score was >3, corresponding to a possible, probable, or highly probable causality grading. To achieve a homogeneous cohort, a good approach would have been including only cases with a probable or highly probable causality ranking. The drugs abidol, acetaminophen, oseltamivir, and ribavirin were discussed. For this retrospective study, all data were retrieved from the digital medical records during hospitalization. As a specific appeal, when multiple drugs in combination are used in COVID-19 patients, the RUCAM score is required to evaluate the risk of DILI.
Delgado, 2021 [51] Spain (cases, n = 160) (drugs, n = 18)	The updated RUCAM [6] was used in 124 males and 36 female patients [51], providing in 82/160 patients a probable causality based on a RUCAM score of ≥6 and in 78/160 cases a possible causality ranking based on a RUCAM score of ≥3. The high number of possible causalities could have been avoided by using a prospective study protocol. DILI was defined with serum ALT activity ≥5 times the ULN. During the hospital stay, the mean number of used drugs per patient was 14.7 (SD 7.6), whereby 98.1% received a polypharmacy with >5 drugs. Among the used drugs were acetaminophen, azithromycin, ceftriaxone, dexketoprofen, doxycycline, enoxaparin, hydroxychloroquine, interferon, levofloxacin, lopinavir, metamizole, omeprazole, pantoprazole, piperacillin/tazobactam, remdesivir, ritonavir, and tocilizumab.
Jothimani, 2021 [52] India (cases, n = 1) (drugs, n = 4)	RUCAM was applied without clear definition of the RUCAM version used [5,6] in this male patient with COVID-19 [52], who experienced DILI after using the oral anticoagulant dabigatran, for which a RUCAM score of 7 corresponding to a probable causality was found. Additional drugs included enoxaparin, esomeprazole, and methylprednisolone. It was outlined that the cause of the liver injury is multifactorial in COVID-19.
Kumar, 2021 [53] India (cases, n = 3) (drugs, n = 3)	In this study of three patients (two females, one male) with COVID-19, each was treated with favipiravir that caused DILI, and RUCAM was used without specifying the RUCAM version applied [40]. Likely the updated RUCAM was used, which requires the exclusion of hepatitis E virus (HEV) infection [6], a parameter considered in the present study [53]. HEV is not an element of the original RUCAM [5]. For all three patients, a RUCAM score of 7 was presented consistent with a probable causality [53]. Of note, the second patient also used acetaminophen, and the third patient was also under a treatment with entecavir for his hepatitis B-related cirrhosis, currently with a negative hepatitis B DNA titer.
Yamazaki, 2021 [54] Japan (cases = 1) (drugs = 8)	The updated RUCAM [6] was applied in a male COVID-19 patient experiencing DILI by favipiravir, causing a RUCAM score of 6 in line with a probable causality and not a possible grading as erroneously published [54]. The patient received multimedication, which included interferon-β, lopinavir, meropenem, micafungin, ritonavir, trimethoprim-sulfamethoxazole, and vancomycin. A contributory causal role of vancomycin and meropenem was discussed.
Deng, 2022 [55] China (cases = 2) (drugs 2)	In two patients with COVID-19 [55], the updated RUCAM was used [6], providing with a score of 8 a probable causality for the male patient treated with ibuprofen and with a score of 9 a highly probable causality for the female patient, who used acetaminophen [55]. In three other COVID-19 patients, the LT abnormalities were related to COVID-19 infection. In this study, many other COVID-19 patients were not treated by antiviral drugs.
Naseralallah, 2022 [56] Qatar (cases = 72) (drugs = 8)	A total of 72 COVID-19 patients with DILI in temporal association with the use of acetaminophen, amoxicillin-clavulanate, azithromycin, ceftriaxone, cefuroxime, favipiravir, hydroxychloroquine, and lopinavir were analyzed [56]. With the updated RUCAM [6], causality was excluded in 4.17% of the cases, unlikely in 12.5%, possible in 45.83%, probable in 34.72%, and highly probable in 2.78% of the cases [56]. Azithromycin was the most used drug implicated in causing DILI.

Retrieved from an earlier open access report [57] and updated from a recent publication [58]. Abbreviations: COVID-19, Coronavirus disease-2019; DILI, Drug-induced liver injury; RUCAM, Roussel Uclaf Causality Assessment Method.

**Table 2:** List of drugs most implicated in causing DILI with verified diagnosis using RUCAM to assess causality, modified from a previous report [13].

Drugs	RUCAM-Based DILI Cases (n)
1. Amoxicillin-clavulanate	333
2. Flucloxacilllin	130
3. Atorvastatin	50
4. Disulfiram	48
5. Diclofenac	46
6. Simvastatin	41
7. Carbamazepine	38
8. Ibuprofen	37
9. Erythromycin	27
10. Anabolic steroids	26
11. Phenytoin	22
12. Sulfamethoxazole/Trimethoprim	21
13. Isoniazid	19
14. Ticlopidine	19
15. Azathioprine/6-Mercaptopurine	17
16. Contraceptives	17
17. Flutamide	17
18. Halothane	15
19. Nimesulide	13
20. Valproate	13
22. Nitrofurantoin	11
23. Methotrexate	6
24. Rifampicin	7
25. Sulfazalazine	7
26. Pyrazinamide	5
27. Natriumaurothiolate	5
28. Sulindac	5
29. Amiodarone	4
30. Interferon beta	3
31. Propylthiouracil	2
32. Allopurinol	1
33. Hydralazine	1
34. Infliximab	1
35. Interferon alpha/Peginterferon 1	1
36. Ketoconazole	1

The RUCAM-based DILI cases represent the total number of cases by drug or drug class and were retrieved from the international literature [44,45,66,67,68,69,70,71,72,73,74,75,76,77]. Abbreviations: DILI, drug-induced liver injury; RUCAM, Roussel Uclaf Causality Assessment Method.

**Table 3:** Serum antibodies as immune features of iDILI. Serum antibodies in patients with RUCAM-based iDILI following use of volatile anesthetics.

Immune Parameter	Details of RUCAM-Based iDILI Cases	Drug	First Author
Serum anti-CYP 2E1	Patients with iDILI by the volatile anesthetic sevoflurane showed positive serum titers of anti-CYP 2E1 in cases with highly probable causalities and well-described clinical features including fever, flu-like symptoms, jaundice, vomiting, right upper quadrant abdominal pain, reduced appetite, rash, and myalgias after the second anesthesias. Liver histology showed centrilobular necrosis with hemorrhage as well as rosetting of liver cells.	Sevoflurane	Nicoll, 2012 [106]
Serum anti-CYP 2E1	Detailed clinical description of RUCAM-based iDILI case caused by a combination of volatile anesthetics. Special care was taken considering alternative causes such as hypotension and DILI by antibiotics or paracetamol.	Sevoflurane + desflurane	Bishop, 2019 [107]
Serum anti-TFA	Most exciting, in some patients with RUCAM-based iDILI, trifluoroacetyl (TFA) halide as toxic intermediates were detected, arising from drug metabolism via CYP 2E1 and providing the potential of protein adduct formation and free radical generation, conditions resulting in detectable anti-TFA antibodies.	Sevoflurane + desflurne	Nicoll, 2012 [106] Bishop, 2019 [107]

Abbreviations: CYP, Cytochrome P40; DILI, drug-induced liver injury; RUCAM, Roussel Uclaf Causality Assessment Method.

**Table 4:** Immune features in selected DIAIH cases.

Serum Immune Parameter	Details of RUCAM-Based DIAIH Cases	Selected Drugs	First Author
ALKMA, ANA, ASLA, ASMA	The immuno-allergic phenotype is characterized by any combination of rash, facial edema, lymphadenopathy, fever, and eosinophilia, ranging from 11–27% with jaundice in 70–75%. Typical is an increased IgG level. Histology: portal inflammation, plama cell infiltrates, rosettes, and focal necrosis.	Atorvastatin	Tan, 2022 [124]
		Diclofenac	
		Etanercept	
		Infliximab	
		Methyldopa	
		Minocycline	
		Nitrofurantoin	
		Rosuvastatin	
AMA, ANA, ASMA	At admission, jaundice and rash were typical features. Histology revealed severe portal inflammation, portal plasma cells, rosette formation, and severe focal necrosis.	Antimicrobials	Licata, 2014 [125]; Stephens, 2021 [126]
ANA, ASMA	With 91%, females were predominant. Symptoms and signs included jaundice, itching, rash, fever, and eosinophilia. Most patients had hepatocellular injury.	Hydralazine	de Boer 2017 [127]
		Methyldopa	
		Minocycline	
		Nitrofurantoin	
ALKMA, AMA, ANA, ASMA	Serum antibodies were confirmed. The decrease of serum ALT activities 1 week after initiation of the steroid therapy was more pronounced compared with the genuine AIH that could help differentiate DIAIH from AIH according to authors.	Atorvastatin	Weber, 2019 [128]
		Dabigatran	
		Diclofenac	
		Ezetimibe	
		Metamizole	
		Rivaroxaban	
SMA	Efficacy of immunosuppressive therapy was confirmed.	Infliximab	Valgareisson 2019 [129]
ANA, ASMA	Confirmation of serum antibodies in a perfect study with exclusion of various alternative causes like DILI by antibiotics.	Adalimumab	Shelton, 2015 [130]
		Certolizumab	
		Infliximab	
AMA, ANA ASMA	Effective treatment with steroids and/or immunomodulators. First DIAIH study from Latin America (Colombia).	Nitrofurantoin NSAIDs	Martinez-Casas, 2018 [131]
ANA, ASMA	Prednisone monotherapy or followed by azathioprine combined with azatioprine. No liver-related mortality; no need for liver transplantation.	Diclofenac Nitrofurantoin Statins	Yeong, 2016 [132]
ANA	In Japan, DIAIH is caused by a few chemical drugs but mostly by TCMs.	NSAIDs Clarithromycin	Hisamochu, 2016 [133]

Abbreviations: ALKMA, anti-liver kidney microsomes antibodies; AMA, anti-mitochondrial antibodies; ANA, anti-nuclear antibodies; ASMA, anti-smooth muscle antibodies; ASLA, anti-soluble liver antigen antibodies; DIAIH, drug-induced autoimmune hepatitis; NSAIDs, non-steroidal anti-inflammatory drugs; RUCAM, Roussel Uclaf Causality Assessment Method; TCM, traditional Chinese medicines.

## Data Availability

Data are available in the quoted reports.
